# Exploration of Cytotoxic Potential of Longifolene/Junipene Isolated from *Chrysopogon zizanioides*

**DOI:** 10.3390/molecules27185764

**Published:** 2022-09-06

**Authors:** Madhuri Grover, Tapan Behl, Tarun Virmani, Mohit Sanduja, Hafiz A. Makeen, Mohammed Albratty, Hassan A. Alhazmi, Abdulkarim M. Meraya, Simona Gabriela Bungau

**Affiliations:** 1 Bhawani Shankar (B.S.) Anangpuria Institute of Pharmacy, Alampur 121004, India; 2 School of Pharmaceutical Sciences, Modern Vidya Niketan MVN University, Palwal 121105, India; 3 School of Health Sciences, University of Petroleum and Energy Studies, Dehradun 248007, India; 4 Department of Pharmacy, School of Medical and Allied Sciences, GD Goenka University, Gurugram 122103, India; 5 Pharmacy Practice Research Unit, Clinical Pharmacy Department, College of Pharmacy, Jazan University, Jazan 45142, Saudi Arabia; 6 Department of Pharmaceutical Chemistry, College of Pharmacy, Jazan University, Jazan 45142, Saudi Arabia; 7 Substance Abuse and Toxicology Research Centre, Jazan University, Jazan 45142, Saudi Arabia; 8 Department of Pharmacy, Faculty of Medicine and Pharmacy, University of Oradea, 410087 Oradea, Romania; 9 Doctoral School of Biomedical Sciences, University of Oradea, 410087 Oradea, Romania

**Keywords:** cancer, vetiver, aromatherapy, longifolene, cytotoxicity

## Abstract

Since ancient times, *Chrysopogon zizanioides* has been utilized as a traditional medicinal plant for the treatment of numerous ailments, but neither its plant extract form nor its phytoconstituents have been fully explored. With this in mind, the present research was designed to isolate and structurally characterize one of its chemical constituents and evaluate its cytotoxic potential. Therefore, an ethanolic extract of roots was prepared and subjected to column chromatography using solvents of varying polarities. The obtained pure compound was characterized using various chromatographic and spectroscopic techniques such as high-performance liquid chromatography (HPLC), carbon and proton nuclear magnetic resonance (NMR), and liquid chromatography–mass spectroscopy (LC-MS) and identified as longifolene. This compound was evaluated for its cytotoxic potential using an MTT (3-(4,5-dimethylthiazol-2-yl)-2,5-diphenyltetrazolium bromide) assay on the prostate (DU-145), oral (SCC-29B) cancer cell line and normal kidney cell line (Vero cells), taking doxorubicin as a standard drug. The obtained outcomes revealed that longifolene possesses cytotoxic potential against both prostate (IC_50_ = 78.64 µg/mL) as well as oral (IC_50_ = 88.92 µg/mL) cancer cell lines with the least toxicity in healthy Vero cells (IC_50_ = 246.3 µg/mL) when compared to doxorubicin. Hence, this primary exploratory study of longifolene exhibited its cytotoxic potency along with wide safety margins in healthy cell lines, giving an idea that the compounds possess some ability to differentiate between cancerous cells and healthy cells.

## 1. Introduction

Cancer is the most perplexing disease of the 21st century and is increasing tremendously without any discrimination based on age, cell, and gender [1,2]. After cardiovascular disease, cancer is considered the second biggest cause of mortality globally [3]. According to GLOBOCAN stats, 19.3 million new cases were registered in 2020 and approximately 10 million mortality was reported, with Asia accounting for 58.3 percent of cancer fatalities [4]. Though there are multiple treatment options available for treating this disease, none of them provides a 100% cure. Among these treatment options, medicinal plants serve an important role either by providing medicine such as Paclitaxel, Vincristine, and Vinblastine or by alternative treatment options for treating cancer-linked complications such as aromatherapy. The availability of versatile phytochemical constituents accounts for plants’ medicinal value with the least side effects [5,6]. Several types of research have reported that herbs are one the best sources for the supply of natural anti-cancer agents. In the past two decades, a variety of phytoconstituent such as coumarins, curcumin, piperine, lupeol, rutin, betulinic acid, etc., has revealed their efficacy against cancerous cells [7,8,9,10,11]. Still, the hunt for novel cytotoxic chemicals from medicinal plants with anti-proliferative action has been a constant focus of research and development [12].

*Chrysopogon zizanioides* or vetiver belongs to the family Poaceae with around 11,337 more species. It is widely distributed in Asia and Subtropical Asia. It is an evergreen plant with tallness of around 1–1.5 m and roots up to 3 m. It was previously grown to protect the land from various environmental issues such as drought, soil erosion, floods, wastewater management, etc. [13]. However, later its roots become the main center of attention due to their multiple utilities, such as therapeutic, flavoring, perfumery, etc. Its uses are well documented in Ayurveda, Charaka Samhita, and Sushruta Samhita. Therapeutically, it is used for treating ailments such as urinary calculi, dysuria, spermatorrhoea, depression, cardiovascular diseases, git issues, and respiratory disorders (asthma, cough, and tuberculosis) [14,15,16]; for flavoring, where it is used in many foods and beverages; and for perfumery in various flowers, decorations, and aromatherapy [17]. Though its roots are enriched with many phytoconstituents that provide them with versatile usage; unfortunately, the scientific literature still lacks the extensive exploration and documentation of its hidden potential.

Earlier research has documented that an ethanolic extract of vetiver was found to contain 107 compounds, out of which only a few have been explored for their potential and others remain still unknown [18]. As a result, the present research was designed to explore the cytotoxic potential of one of its phytoconstituents using an in vitro cytotoxicity assay, in the hopes of attracting the attention of other scientists to this plant and its roots. Although the vetiver oil finds its utilization in aromatherapy, which deals with reducing cancer-linked complications by blending with other oils, it was not explored for its cytotoxicity.

## 2. Results and Discussion

### 2.1. Percent Extractive Yield Value

An extract contains versatile phytochemical constituents such as alkaloids, flavonoids, tannins, saponins, etc., which are found to be responsible for multiple pharmacological activities. Based upon the presence of different phytoconstituents, the method of extraction can be selected, which further affects the yield value too.

The following formula can be used to calculate the percent extractive yield value of an extract:

% yield = Amount of extract obtained × 100/Total amount of roots taken(1)

From 100 g roots, a percentage yield of 1.5 g was produced (1.5% *w*/*w*).

### 2.2. Isolation of a Compound from Chrysopogon zizanioides

The crude extract was subjected to column chromatography and 4 bands were obtained, which were eluted and collected using a mobile phase. The band with the highest color intensity was repetitively purified by preparative thin-layer chromatography to obtain the purified compound, whose R_f_ value was found to be 0.79. The purified compound was obtained as a little yellowish and oily, which was further structurally characterized. The yield obtained from 300 g roots was 2.64 mL, and from 100 g, it was 0.88 mL.

### 2.3. Structural Characterization of Isolated Unknown Compound ‘X’

#### 2.3.1. HPLC Analysis of Isolated Unknown Compound ‘X’

The HPLC profile of an isolated unknown compound was carried out to analyze the purity profile of X. The obtained chromatogram exhibited a single major peak with an area percentage of 99.1, at a retention time of 2.133 min, while the other peak was at 1.807 min with an area percentage of 0.9, which confirmed that the obtained compound was pure enough to proceed with the structural characterization and cytotoxicity profile studies. The HPLC profile of the isolated unknown compound is shown in Figure S1.

#### 2.3.2. FTIR of Isolated Unknown Compound ‘X’

**Figure 1:**
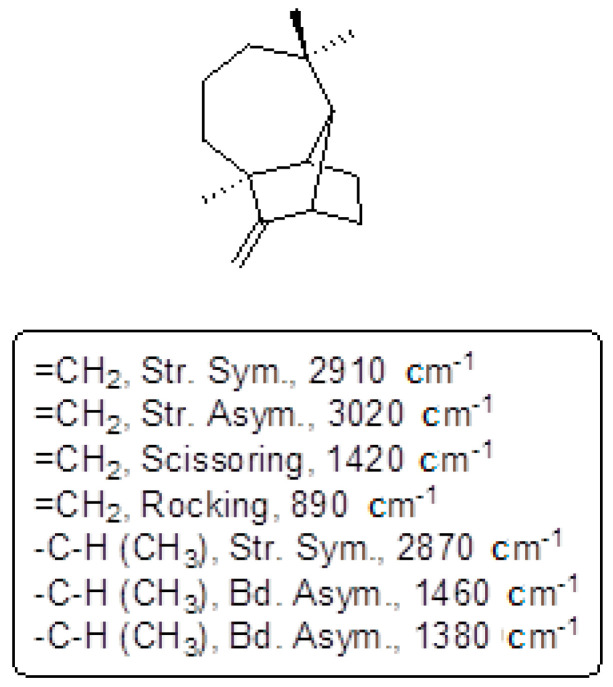
Wave numbers of different functional groups of isolated compound ‘X’.

The FTIR spectrum of the purified compound X has shown the presence of different groups, which is revealed in Figure S2 (Supplementary Materials). Each of its values is found in conformity with the structure of longifolene which are listed below in Figure 1.

#### 2.3.3. NMR of Isolated Unknown Compound ‘X’

##### Proton NMR

The ^1^H NMR spectrum of the purified unknown compound X showed the peak δ at 0.8 to 4.73, which revealed that the compound is aliphatic. The obtained peaks are listed below and the spectra are shown in Figure S3.

Obtained peaks: 4.73 (1H, singlet, =CH), 4.55 (1H, singlet, =CH), 2.5 (1H, singlet, -CH-),1.51–1.75 (6H, multiplet, -CH_2_-), 1.2–1.49 (5H, multiplet, -CH_2_-), 0.9–1.1(8H, multiplet, -CH_3_, -CH_2_-), 0.87 (3H, singlet, -CH_3_).

##### Carbon 13 ^13^C NMR

The ^13^C NMR spectrum of the isolated unknown compound X showed the peak δ from 20.56 to 123.92. The observed peaks are listed below and shown in Figure S4.

^13^C NMR (d_6_– DMSO, 400 MHz, δ, TMS = 0): 20.56, 25.04, 29.14, 29.80, 30.20, 30.38, 33.24, 35.93, 42.74, 43.53, 44.33, 47.21, 61.31, 99.09, and 167.

All the interpreted peaks were also confirmed through the reported literature [19].

#### 2.3.4. LC-MS of Isolated Unknown Compound ‘X’

The MS-ESI spectrum of the purified unknown compound X showed a peak at *m*/*z* 204.6 (M+) exhibiting the molecular weight of the isolated compound (Figure S5). MS-ESI (calculated): m/z 204.36 MS-ESI (experimental): *m*/*z* 204.6.

**Figure 2:**
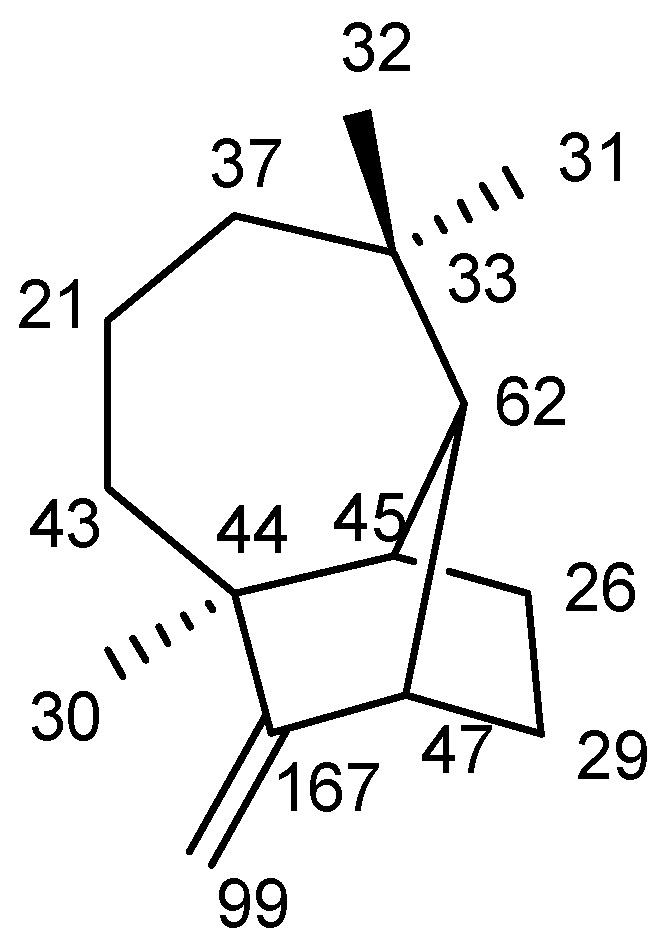
Two-dimensional structure of longifolene/junipene with NMR results.

Based on the results obtained from FTIR, ^1^H and ^13^C NMR, and LC-MS, the structure is confirmed as longifolene/junipene and its details are as follows:

Name of compound: longifolene/junipene
Molecular formula: C_15_H_24_
Molecular weight: 204.6
Its structure is mentioned in Figure 2.

#### 2.3.5. Cytotoxicity Profile of Isolated Junipene/Longifolene and Doxorubicin

**Figure 3:**
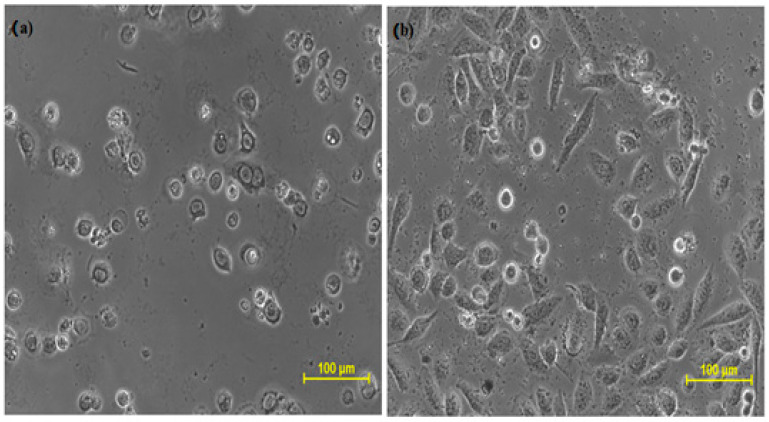
Cytotoxic effect of (**a**) doxorubicin and (**b**) longifolene on DU-145 cells.

**Figure 4:**
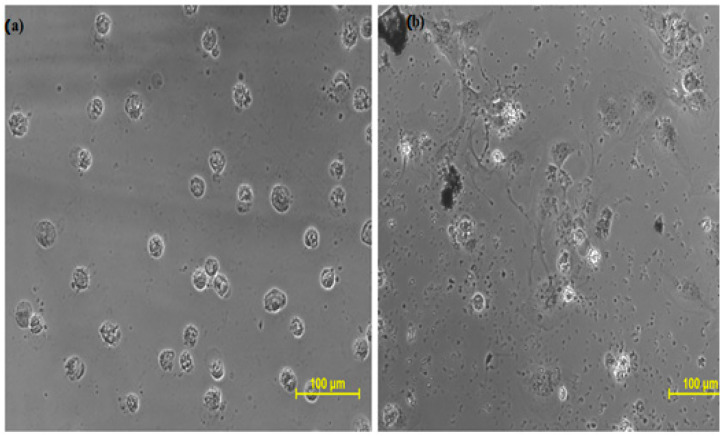
Cytotoxic effect of (**a**) Doxorubicin and (**b**) longifolene on SCC-29B cells.

**Figure 5:**
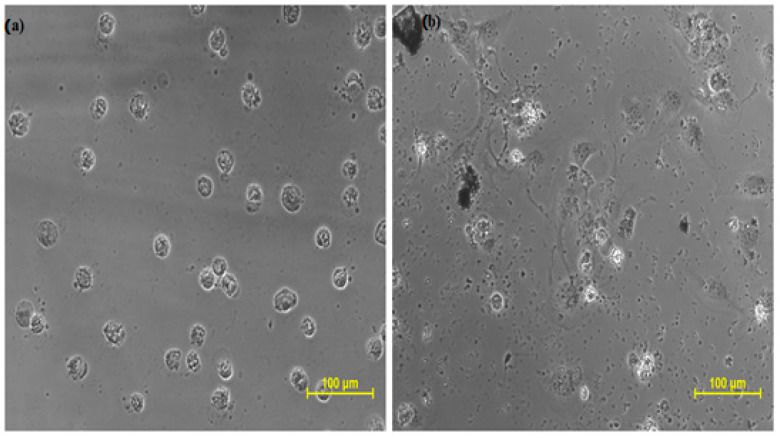
Effect of Doxorubicin and longifolene on Vero cells (normal kidney cell line): (**a**) Vero cells treated with Doxorubicin (**b**) Vero cells treated with longifolene.

The cytotoxicity profile of isolated longifolene was explored for the very first time on the given cell lines by MTT assay, taking doxorubicin as the standard drug. This assay was performed at SKANDA Life Sciences, Bangalore, and the IC_50_ values are reported in Table 1, Table 2, Table 3 and Table 4 and Figure 3, Figure 4 and Figure 5.

The aforementioned results were statistically analyzed using one-way ANOVA, and the obtained f-stat value was 3.77 and the *p*-value was 0.124. As the obtained f-stat value is more than the tabulated value (α = 0.05), so we reject the null hypothesis of ANOVA and concluded that the two samples exhibited statistically significant outcomes. The results exhibited that the isolated compound longifolene exhibited cytotoxicity against both kinds of cancer cell lines but was more sensitive toward DU-145 than SCC-29B. Furthermore, when the cytotoxic profile was compared with the standard drug doxorubicin, it was determined to be substantially safer for the healthy Vero cell line confirming the safety of non-cancerous cells. The safety margins allow for an increase in the concentration of longifolene thrice without any effect on the healthy cell lines. We know that almost all potent chemotherapeutic agents possess good cytotoxicity, but they lack the differentiation between cancerous cells and non-cancerous cells. This inability of distinction makes the patient suffer from many side effects such as baldness, weight loss, loss of appetite, weak immunity, etc.

Its safety profile on human health is also reported by Api et al., in 2019. He reported that longifolene does not cause any genotoxicity, repeated dose toxicity, developmental and reproductive toxicity, or phototoxicity on human health [20].

Another study by Zhu et al., (2020) reported the cytotoxicity profile of seventeen longifolene-derived tetralone derivatives bearing 1,2,4-triazole moiety, amongst which few compounds were found to be comparable or even with more efficient cytotoxicity than 5-Fluorouracil. This study concludes that few alterations in the chemical structure of longifolene can give comparable or better results than the already existing chemotherapeutic agents [21].

The differentiation between cancerous and non-cancerous cells could be attributed to some mechanism that can separately identify the two types of cells based on their proliferative activity. Similar results were reported by Li et al., (2022) about potent novel longifolene-derived tetralin pyrimidine compounds that exhibited the least cytotoxicity toward human normal liver cells L02 [22]. Another study revealed that sesquiterpenes exhibit cytotoxic activity by inhibiting TNF-α, IL-1 [23], Farnesyltransferase, tubulin proliferation [24], G2/DNA [25], and lipase [26], as longifolene is also a sesquiterpene so a similar mechanistic pathway can also be explored for it.

## 3. Experimental Section

### 3.1. Materials

For carrying out the research study, all the chemicals were purchased from Sigma-Aldrich, Loba, and CDH, India, and taken into use without further purification.

### 3.2. Plant Roots and Their Extraction

For the execution of the present research work, the roots of *Chrysopogon zizanioides* were purchased in July 2018 from Khari Baoli, Delhi, India, and validated by emeritus scientist Dr Sunita Garg and Mr Jayasomu, senior principal scientist at NISCAIR Delhi, with voucher no. NISCAIR/RMD/Consult/2018/3239-40-1. A specimen sample was also submitted to RHMD, NISCAIR as a sample. Afterward, accurately weighed 100 g of the roots were cleaned, dried, and grounded [18,27]. The grounded roots were soaked into 1 L of ethanol solvent for extraction by the cold maceration method for one week. Based on the results obtained from the cytotoxic profile of different solvents of *Chrysopogon zizanioides*, ethanol solvent was selected for further isolation of susceptible phytoconstituents [15].

### 3.3. Isolation of a Pure Compound

#### Separation of Bands and Their Fractions

The obtained ethanolic extract was concentrated at an ambient temperature to omit the loss of any phytoconstituent. For isolation, this concentrated extract was mixed with purified silica gel (1:8) to prepare the slurry. Afterward, the obtained slurry was chromatographed by taking silica gel as stationary phase (60–120 mesh, 1500 g) and hexane: chloroform (85:15) as mobile phase (validated using TLC). The mobile phase was run initially in a gradient way and the fraction of each band was collected (200 mL). A total of four different bands (V_1_–V_4_) were also detected on TLC (SiO_2_) using vanillin–sulfuric acid as a spray reagent. Out of four bands, the color intensity of third band V_3_ (eluted at third position) was found to be maximum. Assuming to have a higher yield of phytoconstituent, V_3_ was selected for in-depth investigation. The fraction V_3_ was found to be a complex mixture that further resolved into three bands (V_3a_, V_3b_, V_3c_) on preparative thin-layer chromatography (PTLC) using mobile-phase hexane: CHCl_3_: methanol (65:34:1). The band obtained was eluted with CHCl_3_ (100%) to yield three subfractions from V_3a_ to V_3c_.

##### Isolation of Unknown Compound X

**Figure 6:**
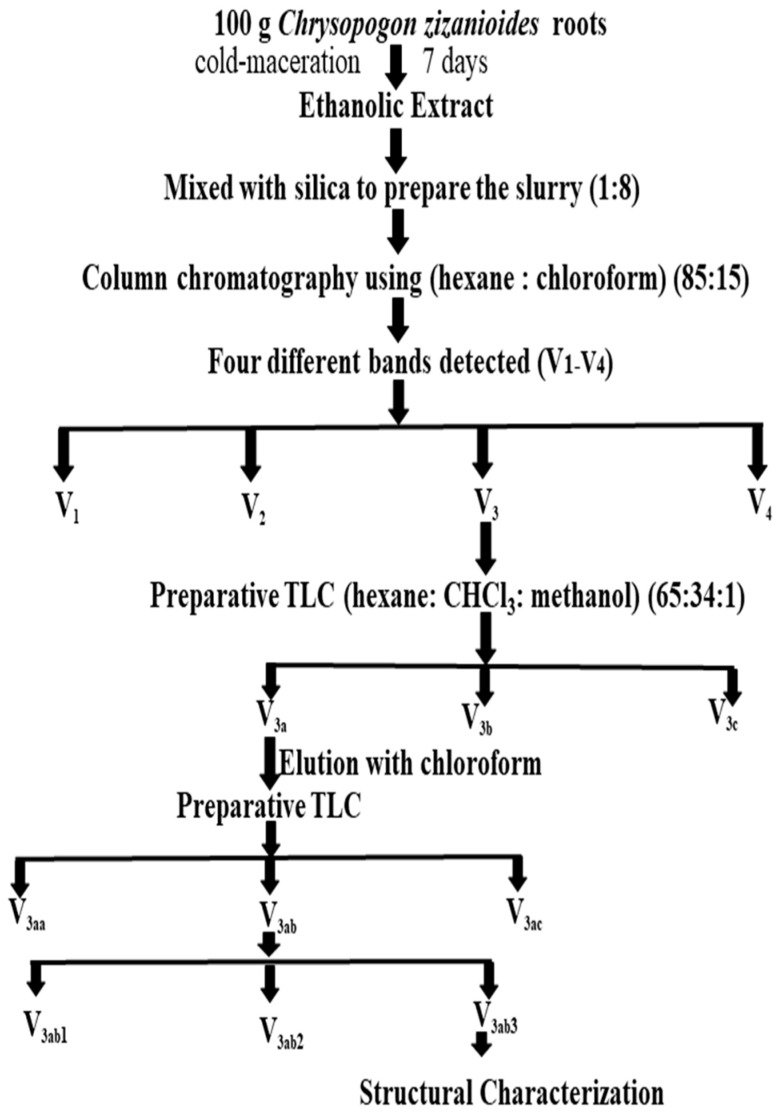
Isolation of compound ‘X’ from *C. zizanioides*.

The intense brightened band V_3a_ was further purified (93 mg) using PTLC, which again resolved into three bands on PTLC. Out of these three bands (V_3aa_, V_3ab_, V_3ac_), the middle one V_3ab_ was further resolved using PTLC, which ultimately yielded a further three constituents (V_3ab1,_ V_3ab2,_ V_3ab3_). The most intense constituent was eluted using CHCl_3_ and selected for further analysis. The isolated compound V_3ab3_ was subjected to MS, NMR, and IR analysis, for structural confirmation. Its isolation procedure is described in Figure 6.

### 3.4. Structural Characterization

All these spectroscopic analyses were carried out at SIMA Labs Pvt. Ltd., Delhi, India, and SICART LAB Pvt. Ltd., Gujarat, India.

#### 3.4.1. HPLC Analysis

Under simple conditions, the isolated unknown compound X was evaluated using the reverse phase HPLC technique in a Shimadzu LC-Prominence 20AT, Japan equipment fitted with a C18 HPLC column from Alltech Associates. A volume of 10 µL sample was filtered and processed for analysis using 80:20:0.1 acetonitrile (MeCN), water, and phosphoric acid (HPLC grade) solvent system, wavelength 425 nm, flow rate1.0 mL/min, and temperature of 35 °C.

#### 3.4.2. Infra-Red (IR) Spectroscopy

The liquid sample of an unknown isolated compound was mixed with solvent Nujal mull for the analysis. The spectroscopy was performed on an IR 400 Spectrophotometer (Shimadzu, Kyoto, Japan) [28] in a wavelength ranging from 400 to 4000 cm^−1^ and resolution of 4 cm^−1^.

#### 3.4.3. Nuclear Magnetic Resonance (NMR)

The proton (^1^H) and carbon (^13^C) NMR spectra of the unknown compound X were carried on Supercon NMR Spectrometer from Bruker, West Germany. The analysis was carried out by mixing the sample in D_2_O and then operating at 500 and 125.7 MHz, respectively. The obtained spectra were interpreted to obtain the structure of the compound.

#### 3.4.4. Mass Spectrometry (MS)

The mass of the sample was revealed using TSQ Quantum Access MAX Triple Quadruples LCMS2010A (advanced version), Shimadzu, Japan. This instrument analyzes polar as well as non-polar compounds with maximum weights up to 2000. For performing this technique, 5 μL sample was injected and analyzed in a range of 50–1050 daltons after setting some parameters such as curtain gas 10, gas 1(20), and needle voltage 5000 V. The obtained molecular peak was noted in the spectra.

### 3.5. Cytotoxic Studies

#### 3.5.1. Cancer Cell Lines

All three cell lines, prostate cancer cell line (DU-145), oral cancer cell line (SCC-29B), and healthy cell line (Vero cells), were procured from American Type Culture Collection (ATCC, Rockvilled, MD, USA) for the cytotoxicity assay. The stock cells were grown in DMEM (Dulbecco’s Modified Eagle Medium) (ATCC, Rockvilled, MD, USA) at 37 °C in a humidified environment of 5% CO_2_. The cytotoxicity of experimental drugs (longifolene and doxorubicin) were checked on these cell lines using an MTT assay at different concentrations:

Standard drug (Doxorubicin): 1.7 µg/mL, 3.4 µg/mL, 6.8 µg/mL, 13.6 µg/mL, 27.16 µg/mL, and 54.32 µg/mL [19].

Test drug (longifolene): 10 µg/mL, 20 µg/mL, 40 µg/mL, 80 µg/mL, 160 µg/mL, and 320 µg/mL [14].

#### 3.5.2. MTT (3-(4,5-Dimethylthiazol-2-yl)-2,5-Diphenyltetrazolium Bromide) Assay

**Figure 7:**
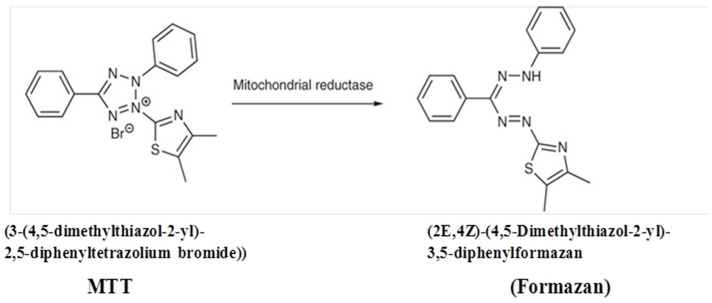
Conversion of MTT dye to Formazan.

An MTT assay mainly employs MTT dye, which is converted to purple formazan by mitochondrial dehydrogenase enzymes of viable cells (Figure 7) that turns the solution purple in color with the addition of DMSO, acidified isopropranolol, etc. The colored solution is spectrophotometrically measured indicating the effect produced by the test and standard sample to provide the result in the form of IC_50_. This MTT assay was carried out at SKANDA Life Sciences Private Limited, using a CO_2_ incubator and Spectramax I3X Plate reader, United States.

Using an appropriate medium containing 10% FBS, the cell density was adjusted to 5.0 × 10^5^ cells/mL density, or 100 µL was added to each well of the 96-well microtiter plate. The supernatant was discarded after 24 h, and the generated layer was rinsed with media. Then, 100 µL of longifolene (varying concentrations) was added to the microtiter plates and the plates were incubated at 37 °C and 5% CO_2_. After the completion of 24 h, 100 µL of MTT (0.5 mg/mL) was added to each well and incubated for 4 h again at 37 °C and 5% CO_2_. The supernatant was discarded once more, and 100 µL DMSO was added to solubilize the formazan that had formed. At a wavelength of 590 nm, the absorbance was measured and the % growth inhibition was computed using Equation (1).

% Inhibition = ((OD of Control − OD of sample)/OD of Control) × 100 (2)

where OD represents the optical density of control.

The IC_50_ value was determined using the sigmoid dose-response curves, and curve fitting method and computed using Graph Pad Prism 6 software, Version 5.03 (Windows), California USA at SKANDA Life Sciences, Bangalore, India. The obtained values were statistically analyzed for their significance using one-way ANOVA.

## 4. Conclusions

The current research documented for the first time the isolation procedure of longifolene from *Chrysopogon zizanioides* and evaluated its cytotoxic potential too. The results obtained indicated that longifolene possesses more cytotoxic potential against the prostate cancer cell line (DU-145) than the oral cancer cell line (SCCC-29B) with a high safety margin in healthy cells. However, if we compare the IC_50_ values of longifolene with doxorubicin, there is a huge gap, but its safety in healthy cell lines provides a great advantage. The present finding of this research revealed that longifolene possesses a natural inclination to develop as a safe anti-neoplastic drug with high efficacy. Moreover, in vivo studies can be taken up in the future for obtaining more reliable and standardized results. Furthermore, the above outcomes provide scientific evidence for utilizing natural compounds as anticancer agents.

## Figures and Tables

**Table 1:** MTT assay of doxorubicin and longifolene on DU-145.

DU-145				
Test Sample	Concentration (µg/mL)	Optical Density at 590 nm	% Inhibition	IC_50_ (µg/mL)
Control	0	0.656	0.00	
Doxorubicin	1.7	0.523	20.27	10.67
	3.4	0.413	37.04	
	6.8	0.365	44.36	
	13.6	0.241	63.26	
	27.16	0.168	74.39	
	54.32	0.069	89.48	
Longifolene	10	0.578	11.89	78.64
	20	0.501	23.63	
	40	0.423	35.52	
	80	0.306	53.35	
	160	0.227	65.40	
	320	0.109	83.38	

**Table 2:** MTT assay of doxorubicin and longifolene on SCC-29B.

SCC-29B				
Test Sample	Concentration (µg/mL)	Optical Density at 590 nm	% Inhibition	IC_50_ Value (µg/mL)
Control	0	0.521	0.00	0
Doxorubicin	1.7	0.423	18.81	9.56
	3.4	0.336	35.51	
	6.8	0.284	45.49	
	13.6	0.206	60.46	
	27.16	0.146	71.98	
	54.32	0.067	87.14	
Longifolene	10	0.468	10.17	88.92
	20	0.417	19.96	
	40	0.346	33.59	
	80	0.275	47.22	
	160	0.198	62.00	
	320	0.109	79.08	

**Table 3:** MTT assay of doxorubicin and longifolene on Vero cell line.

Vero				
Test Sample	Concentration (µg/mL)	Optical Density at 590 nm	% Inhibition	IC_50_ Value (µg/mL)
Control	0	0.498	0.00	0
Doxorubicin	1.7	0.467	6.22	29.12
	3.4	0.429	13.86	
	6.8	0.389	21.89	
	13.6	0.352	29.32	
	27.16	0.260	47.79	
	54.32	0.189	62.05	
Longifolene	10	0.486	2.41	246.3
	20	0.456	8.43	
	40	0.410	17.67	
	80	0.369	25.90	
	160	0.320	35.74	
	320	0.213	57.23	

**Table 4:** Cytotoxicity profile with IC_50_ (μg/mL) values of longifolene and doxorubicin.

Cell Type	Cell Line	Cytotoxicity Profile (IC_50_ µg/mL)	
		Longifolene (µg/mL)	Dox (µg/mL)
Prostate Cancer	DU-145	78.64 ± 0.5	10.67 ± 0.5
Oral Cancer	SCC-29B	88.92 ± 0.5	9.56 ± 0.5
Healthy cell line	Vero cells	246.3 ± 0.5	29.12 ± 0.5

Where, Dox = Doxorubicin, ±0.5 = standard error mean.

## Data Availability

Not applicable.

## Supplementary Materials

The following supporting information can be downloaded at: https://www.mdpi.com/article/10.3390/molecules27185764/s1, Figure S1: HPLC analysis of isolated unknown compound ‘X’, Figure S2: FTIR of the standard and isolated unknown compound ‘X’, Figure S3: Proton [^1^H] NMR of isolated unknown compound ‘X’, Figure S4. Carbon [^13^C] NMR of isolated unknown compound ‘X’, Figure S5. LC-MS of isolated unknown compound ‘X’.
