# Supplementary material for: Exploration of Cytotoxic Potential of Longifolene/Junipene Isolated from Chrysopogon zizanioides
Source: Molecules. 2022 Sep 6;27(18):5764. doi: 10.3390/molecules27185764 (PMC9504982; doi:10.3390/molecules27185764)
Supplement: Supplementary file 1 [file molecules-27-05764-s001.zip › molecules-1872699-supplementary.pdf]

Supplementary Materials

Exploration of Cytotoxic Potential of Longifolene/Junipene Isolated from *Chrysopogon zizanioides*

Madhuri Grover [1,2], Tapan Behl [3,*], Tarun Virmani [2], Mohit Sanduja [4], Hafiz A. Makeen [5], Mohammed Albratty [6], Hassan A. Alhazmi [6,7], Abdulkarim M. Meraya [5] and Simona Gabriela Bungau [8,9,*]

[1] Bhawani Shankar (B.S.) Anangpuria Institute of Pharmacy, Alampur, Ballabgarh 121004, India
[2] School of Pharmaceutical Sciences, Modern vidya Niketan MVN University, Palwal 121105, Haryana, India
[3] School of Health Sciences, University of Petroleum and Energy Studies, Dehradun 248007, Uttarakhand, India
[4] Department of Pharmacy, School of Medical and Allied Sciences, GD Goenka University, Gurugram 122103, Haryana, India
[5] Pharmacy Practice Research Unit, Clinical Pharmacy Department, College of Pharmacy, Jazan University, Jazan 45142, Saudi Arabia
[6] Department of Pharmaceutical Chemistry, College of Pharmacy, Jazan University, Jazan 45142, Saudi Arabia
[7] Substance Abuse and Toxicology Research Centre, Jazan University, Jazan 45142, Saudi Arabia
[8] Department of Pharmacy, Faculty of Medicine and Pharmacy, University of Oradea, 410087 Oradea, Romania
[9] Doctoral School of Biomedical Sciences, University of Oradea, 410087 Oradea, Romania
* Correspondence: tapanbehl31@gmail.com (T.B.); simonabungau@gmail.com (S.G.B.)

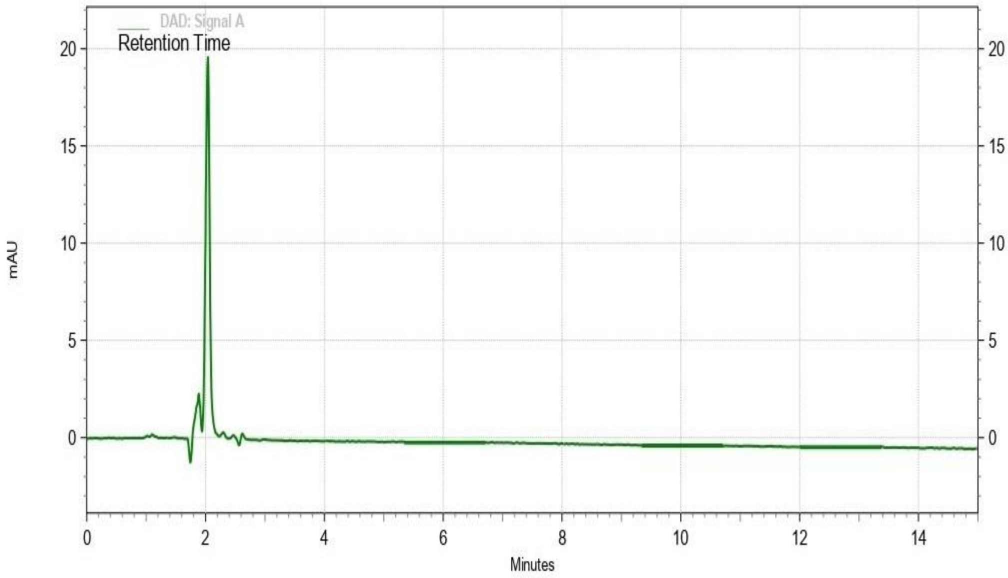

Figure S1. HPLC analysis of isolated unknown compound 'X'.

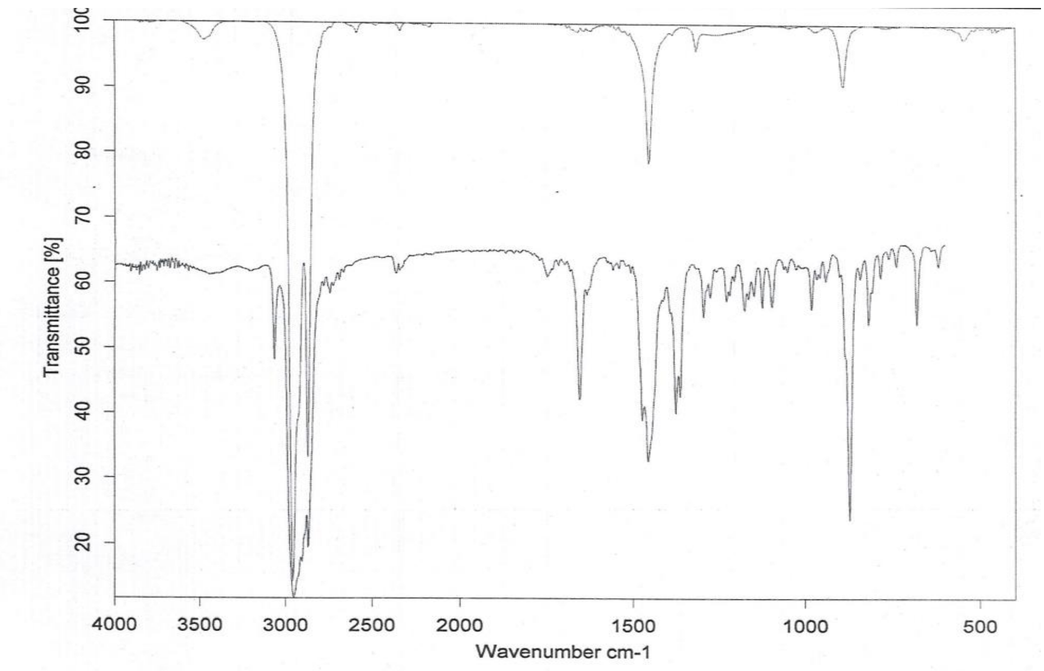

Figure S2. FTIR of the standard and isolated unknown compound 'X'.

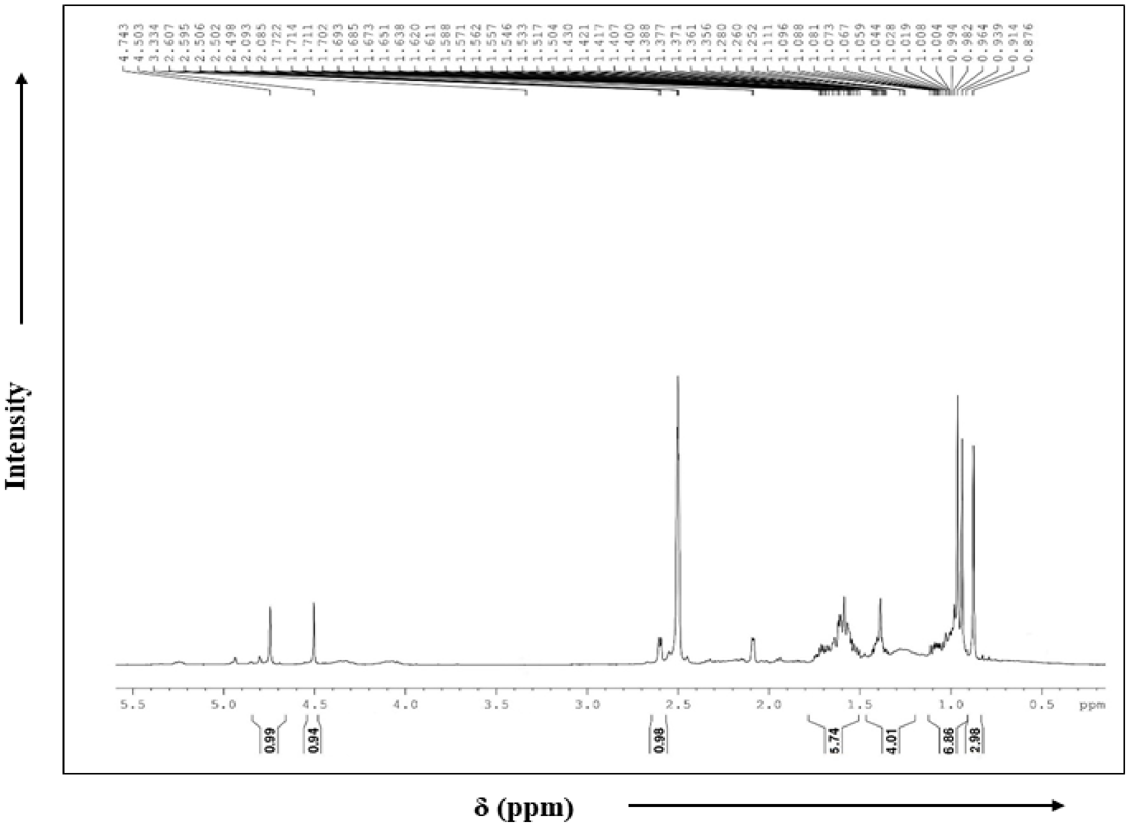

Figure S3. Proton [1H] NMR of isolated unknown compound 'X'.

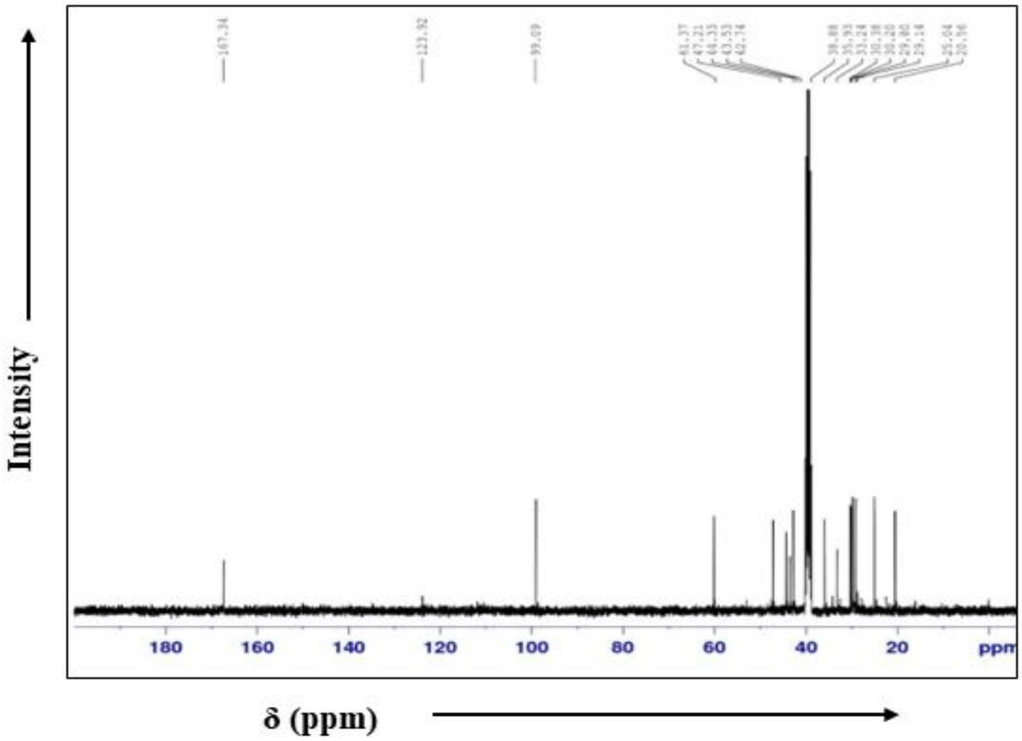

Figure S4. Carbon [^{13}C] NMR of isolated unknown compound 'X'.

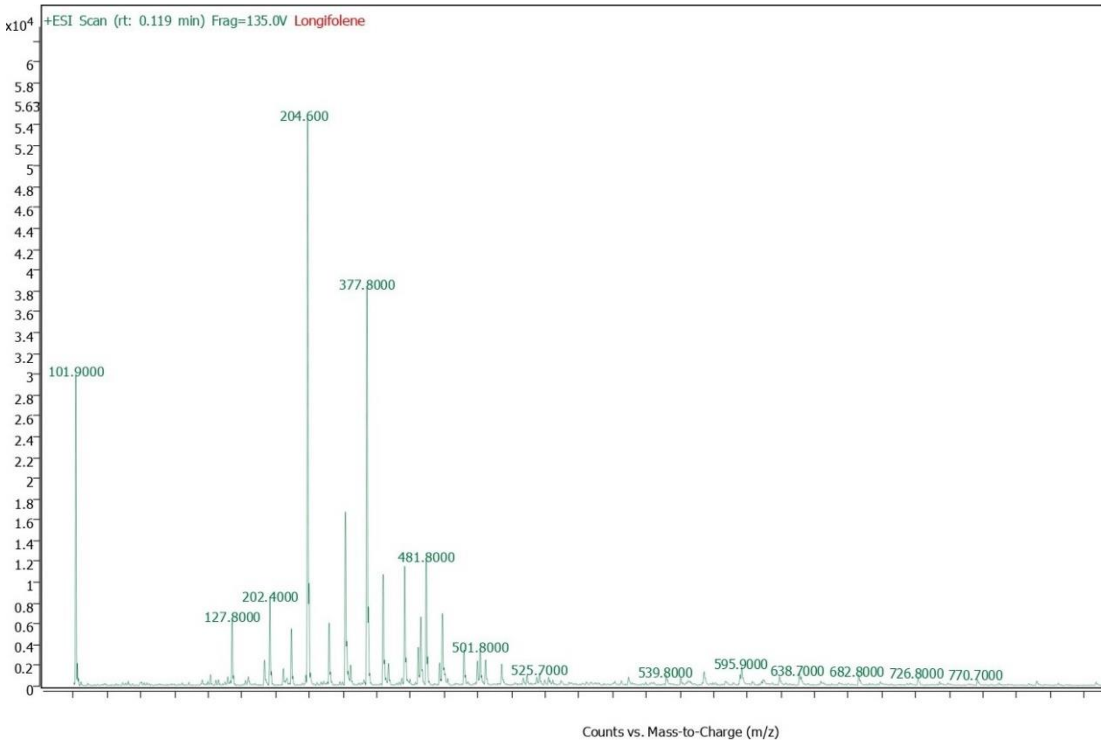

Figure S5. LC-MS of isolated unknown compound 'X'.
